# Intestinal Autophagy Improves Healthspan and Longevity in *C*. *elegans* during Dietary Restriction

**DOI:** 10.1371/journal.pgen.1006135

**Published:** 2016-07-14

**Authors:** Sara Gelino, Jessica T. Chang, Caroline Kumsta, Xingyu She, Andrew Davis, Christian Nguyen, Siler Panowski, Malene Hansen

**Affiliations:** 1 Program of Development, Aging and Regeneration, Sanford Burnham Prebys Medical Discovery Institute, La Jolla, California, United States of America; 2 Graduate School of Biomedical Sciences, Sanford Burnham Prebys Medical Discovery Institute, La Jolla, California, United States of America; 3 Molecular and Cell Biology Laboratory, The Howard Hughes Medical Institute, The Glenn Center for Aging Research, The Salk Institute for Biological Studies, La Jolla, California, United States of America; Stanford University Medical Center, UNITED STATES

## Abstract

Dietary restriction (DR) is a dietary regimen that extends lifespan in many organisms. One mechanism contributing to the conserved effect of DR on longevity is the cellular recycling process autophagy, which is induced in response to nutrient scarcity and increases sequestration of cytosolic material into double-membrane autophagosomes for degradation in the lysosome. Although autophagy plays a direct role in DR-mediated lifespan extension in the nematode *Caenorhabditis elegans*, the contribution of autophagy in individual tissues remains unclear. In this study, we show a critical role for autophagy in the intestine, a major metabolic tissue, to ensure lifespan extension of dietary-restricted *eat-2* mutants. The intestine of *eat-2* mutants has an enlarged lysosomal compartment and flux assays indicate increased turnover of autophagosomes, consistent with an induction of autophagy in this tissue. This increase in intestinal autophagy may underlie the improved intestinal integrity we observe in *eat-2* mutants, since whole-body and intestinal-specific inhibition of autophagy in *eat-2* mutants greatly impairs the intestinal barrier function. Interestingly, intestinal-specific inhibition of autophagy in *eat-2* mutants leads to a decrease in motility with age, alluding to a potential cell non-autonomous role for autophagy in the intestine. Collectively, these results highlight important functions for autophagy in the intestine of dietary-restricted *C*. *elegans*.

## Author Summary

Dietary restriction (DR) without inducing malnutrition has robust beneficial effects on lifespan in many species, including humans. The cellular recycling process of autophagy contributes to DR-mediated longevity. Autophagy is triggered by nutrient scarcity and increases the degradation of cytosolic molecules and organelles in the lysosomes. Using the nematode *Caenorhabditis elegans* as a model organism, we previously showed that genes involved in autophagy are required for lifespan extension through DR; however, it is not clear whether autophagy in individual tissues plays critical roles in DR-mediated longevity. Here, we investigated the contribution of autophagy in genetically dietary-restricted *eat-2* mutants. Our major findings include: (i) Inhibition of autophagy in the intestine prevents the long lifespan observed in *eat-2* mutants; (ii) the intestine of *eat-2* mutants contains an expanded lysosomal compartment and flux assays indicate increased autophagosome turnover, consistent with elevated autophagy in this tissue; (iii) intestinal autophagy is required for the improved intestinal integrity observed in *eat-2* mutants; (iv) autophagy inhibition impairs motility in older animals; and (v) inhibition of autophagy in the intestine accelerates the motility decline in *eat-2* mutants. Collectively, these studies suggest a critical role for intestinal autophagy in dietary-restricted animals, and highlight the importance of this process in maintaining fitness and longevity.

## Introduction

Dietary restriction (DR), defined as limited food intake without malnutrition, is the most robust and conserved intervention currently known to delay aging. Since many physiological effects of DR are evolutionarily conserved, genetically tractable model organisms such as the nematode *C*. *elegans* can be exploited as tools to identify the molecular events underlying DR-mediated lifespan extension [1].

Autophagy is an evolutionarily conserved process induced in response to nutrient deprivation via important metabolic regulators such as the nutrient-sensing kinase TOR. During autophagy, cytoplasmic components are first encapsulated within a double-membrane structure called an autophagosome. This structure subsequently fuses with an acidic lysosome to form an autolysosome, in which the sequestered cargo is degraded by hydrolases [2]. The autophagy process is orchestrated by >30 conserved autophagy proteins (encoded by *ATG* genes), many of which function in complexes at different steps in the process. Of particular note is ATG8, a family of lipid-binding proteins, which play essential roles in autophagosome formation, cargo recruitment, and autophagosome–lysosome fusion [2]. During autophagy, ATG8 proteins get post-translationally modified and inserted into both the inner and outer autophagosomal membranes and GFP-tagged ATG8 proteins are commonly used as markers to assess steady-state levels of autophagosomes in many species [2], including *C*. *elegans*, which contain two ATG8 homologs, LGG-1 and LGG-2 [3].

Autophagy is increasingly recognized to play a critical role in lifespan extension promoted by multiple conserved longevity paradigms [4]. In particular, *C*. *elegans* subjected to DR require autophagy genes for lifespan extension and have increased levels of the ATG8 autophagy marker LGG-1 in their hypodermis [5–8]. These observations suggest a model in which the induction of autophagy by DR, plays a beneficial role in contributing to lifespan extension [4]. However, it is currently unclear if the effects of DR on organismal aging originate from individual tissues. While autophagy is modulated in multiple tissues of dietary-restricted mammals, such as the liver, skeletal muscle, and cardiac muscle of mice [9–13] and rats [14], it remains to be directly tested if tissue-specific changes in autophagy are sufficient to contribute to the improved healthspan and longevity of dietary-restricted animals.

Previous experiments in *C*. *elegans* and *Drosophila* have highlighted an important role for the intestine in several longevity paradigms. Specifically, modulation of the expression of certain genetic determinants specifically in the intestine can influence lifespan [15–18, 40]. It however remains unclear how such genetic interventions may affect physiological functions of the intestine. A key role of the intestinal epithelial layer in all organisms is to form a selective permeability barrier that permits absorption of water and critical solutes while maintaining a defense against potentially toxic substances. Studies in *Drosophila* have shown that the intestinal epithelium in flies, as in mammals, possesses a barrier function. This “intestinal barrier function” declines with age but the rate of decline can be slowed by DR [18–20]. However, it is unknown how DR affects this physiological function of the intestine, and if additional longevity paradigms besides DR can similarly prevent its age-dependent decline.

To study the functional role of the intestine in DR-mediated longevity and learn more about how autophagy contributes to the fitness and aging of animals subjected to DR, we examined the autophagy process in the intestine of dietary-restricted *C*. *elegans*. We used *eat-2(ad1116)* mutants, which carry a mutation that compromises food uptake, thereby providing a genetic model of DR. Notably, specific inhibition of autophagy genes in the intestine was sufficient to prevent the long lifespan of *eat-2(ad1116)* mutants, indicating an important role for autophagy in this organ in the DR longevity paradigm. Consistent with this, the intestine of *eat-2(ad1116)* mutants displayed an enlarged lysosomal compartment and increased turnover of an autophagosomal marker compared to wild-type (WT) animals. Using a novel assay in *C*. *elegans* to measure intestinal barrier function, we found that DR slowed the rate at which intestinal integrity decreased with age, and this protective effect was dependent on intestinal expression of autophagy genes. Similarly, whole-body or intestine-specific autophagy gene knockdown alone caused a reduction in motility, an age-dependent trait, irrespective of the genetic background. In contrast, knockdown of autophagy in the muscle did not cause significant changes in motility, but prevented full lifespan extension. Collectively, our results indicate that inhibition of autophagy in the intestine of dietary-restricted animals prevented lifespan extension and resulted in phenotypes reminiscent of aging, thus highlighting important tissue-specific functions for autophagy that contribute to healthspan and longevity.

## Results

### Autophagy genes are required in the intestine for the longevity of dietary-restricted *eat-2* mutants

In *C*. *elegans*, the *eat-2* gene is essential for pharyngeal pumping, and animals carrying mutations in this gene have a reduced rate of food intake and are longer lived [21]. Such mutants therefore provide a genetic model of DR. Autophagy is required for DR to extend lifespan in *C*. *elegans*, since RNAi of the autophagy-related genes *unc-51/ATG1/Ulk1*, *bec-1/ATG6/Beclin1*, *vps-34*, and *atg-7* shortens the long lifespan of animals with *eat-2* mutations [5, 6, 8], while having a relatively small or non-significant effect on the lifespan of wild-type (WT, N2) animals. However, it is not yet known how autophagy regulation in select tissues contributes to DR-induced longevity.

**Fig 1:**
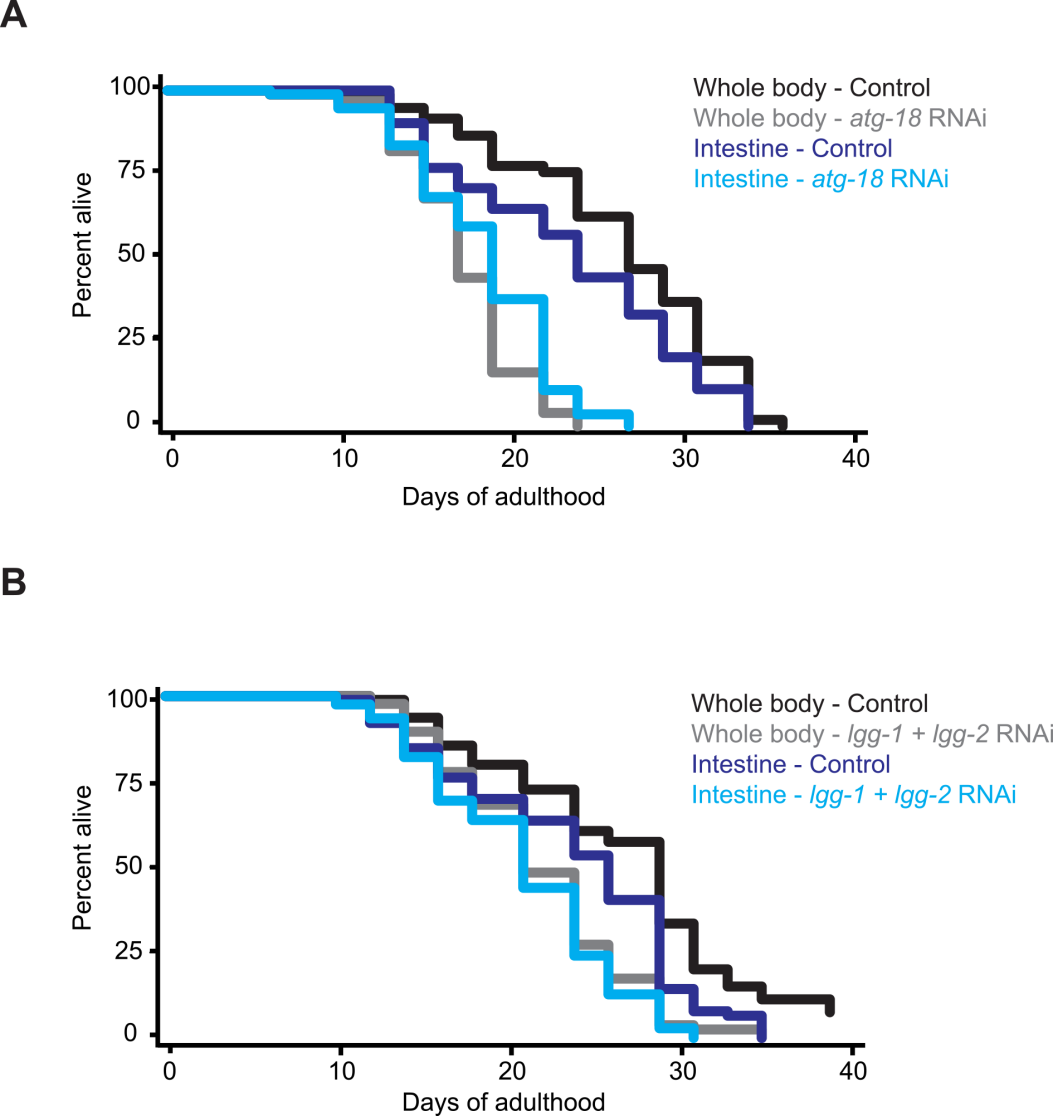
Intestinal-specific autophagy gene knockdown decreases the lifespan of dietary-restricted *eat-2* mutants. Lifespan analysis of *eat-2(ad1116)* single mutants and *eat-2(ad1116)*; *rde-1(ne219)* double mutants expressing *rde-1* from the *nhx-2* intestinal promoter and fed bacteria expressing either empty-vector control or dsRNA encoding either *atg-18*/*WIPI* (**A**) or *lgg-1/ATG8* and *lgg-2/ATG8* (**B**) from Day 1 of adulthood. Dietary-restricted *eat-2* single mutants were subject to whole-body RNAi, whereas *eat-2; rde-1* mutants carrying transgenes were subject to intestinal-specific RNAi. Experiments were carried out at 20°C and were performed at least two times with similar results. See **S1 Table** for details and additional experiments, including in *eat-2; sid-1; gly-19p*::*sid-1* animals.

Since the intestine is a central metabolic tissue key to nutrient uptake and for healthy aging [17], we addressed if intestinal autophagy plays a role in DR-mediated longevity. To inhibit autophagy in the intestine, we utilized tissue-specific RNA interference (RNAi). To this end, we crossed *eat-2(ad1116)* mutants to *rde-1(ne219)* loss-of-function mutants with reconstituted *rde-1* expression from the intestinal *nhx-2* promoter [22]. *rde-1(ne219)* mutants carry a loss-of-function mutation in the *piwi/argonaute* family member essential for the initiation of RNAi [23]. We confirmed the intestinal RNAi specificity of this strain by using RNAi clones targeting different genes expressed inside and outside of the intestine (**S1A Fig**). Lifespan analyses revealed that *eat-2(ad1116); rde-1(ne219)* double mutants, and *eat-2(ad1116)*; *rde-1(ne219)* mutants expressing the *nhx-2*::*rde-1* transgene had lifespans largely comparable to that of *eat-2(ad1116)* single mutants (**Fig 1A and 1B and S1 Table**). These data indicate that neither systemic *rde-1* deficiency nor tissue-specific re-expression of *rde-1* in the intestine significantly affected the lifespan of the *eat-2(ad1116)* mutants.

To determine the effect of intestinal autophagy inhibition on lifespan, we fed the *eat-2(ad1116); rde-1(ne219); nhx-2p*::*rde-1* strain with bacteria carrying empty vector or expressing dsRNA targeting *atg-18/Wipi*, or the two *ATG8* homologs *lgg-1* and *lgg-2* from Day 1 of adulthood. These genes were selected because whole-body inhibition of their expression had potent effects on the lifespan of *eat-2(ad1116)* animals (**S1 Table**). Autophagy gene RNAi caused a significant ~10–22% reduction in the mean lifespan of *eat-2(ad1116); rde-1(ne219)* animals expressing *rde-1* from the *nhx-2* intestinal promoter (**Fig 1A and 1B and S1 Table**). These results were supported by an *eat-2(ad1116)*; *sid-1(qt9)* double mutant expressing *sid-1*, a transmembrane protein that acts as a channel for dsRNA entry [24], from the *gly-19* promoter (**S1 Table**, see also Methods). In parallel to these experiments, we assayed the lifespan of *eat-2(ad1116)* mutants subjected to whole-body RNAi, which generally resulted in stronger lifespan-shortening effects (**Fig 1 and S1 Table**). These observations are consistent with autophagy contributing to DR-mediated lifespan in the intestine and we conclude that autophagy plays an important function in longevity within the intestine of dietary-restricted animals.

### Dietary restriction leads to a decrease in the abundance of autophagosomes in the intestine of *C*. *elegans*

**Fig 2:**
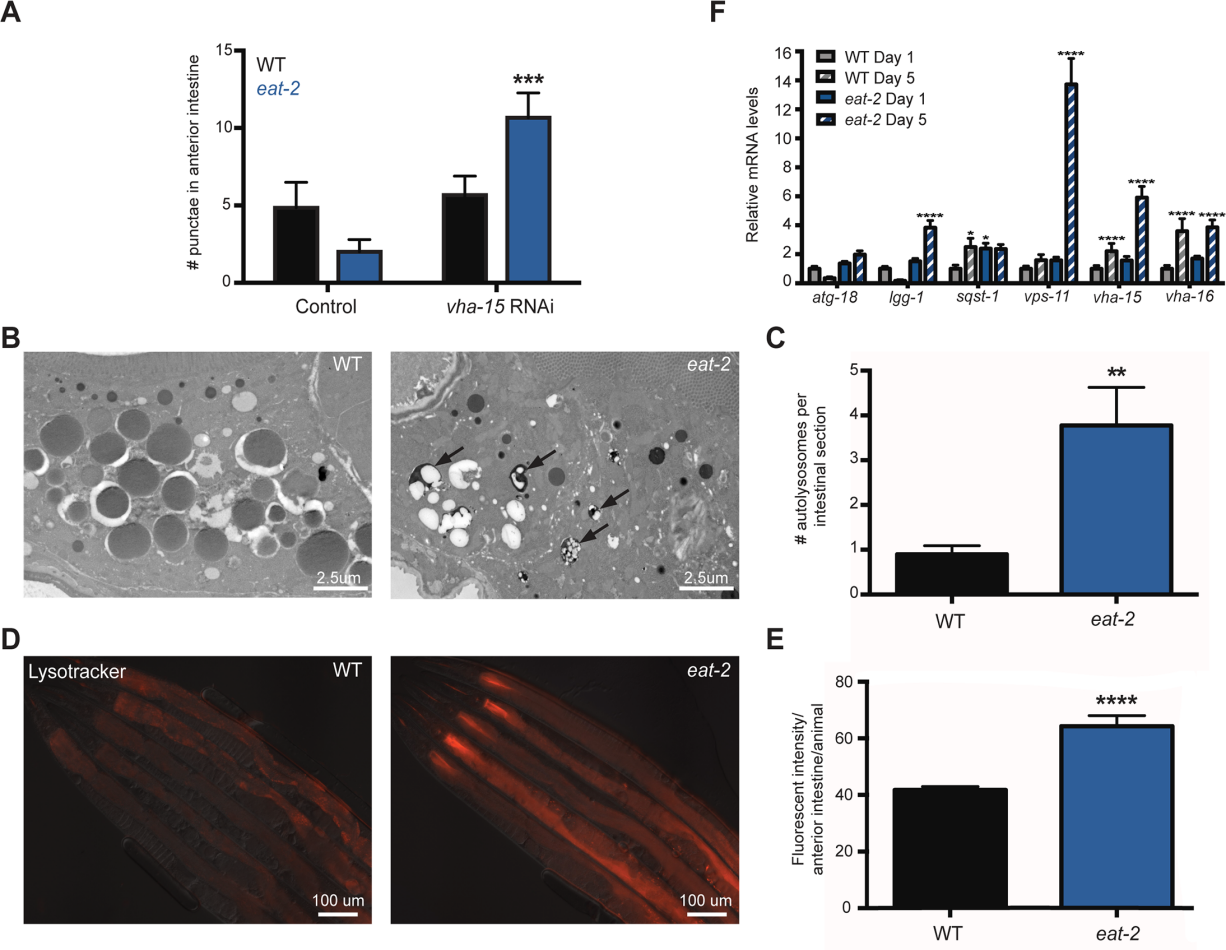
Analysis of the intestine of dietary-restricted *eat-2* mutants indicate enlarged lysosomal compartment and increased autophagic flux. (A) Quantification of GFP::LGG-1 punctae in the anterior intestine of WT and *eat-2(ad1116)* mutants fed from the L4 larval stage to Day 4 of adulthood with bacteria containing empty vector or expressing *vha15*/V-ATPase-targeting dsDNA. Data are the mean ± SEM of 7–21 animals per condition. ***P = 0.0002 for RNAi control *eat-2(ad1116)* versus RNAi for *vha-15*/V-ATPase by two-way ANOVA. The experiment was repeated four times with similar results. (B) Q-PCR analysis of putative autophagy-related and lysosomal gene expression in wild-type (WT) and *eat-2(ad1116)* animals on Days 1 and 5 of adulthood. Data are the mean ± SEM of biological triplicates, each with three technical replicates, and are normalized to expression in the WT (N2) animals. *P<0.05, **P<0.01, ***P<0.001, and ****P<0.0001, one-way ANOVA. (C) Representative electron micrographs of intestinal cross-sections of WT and *eat-2(ad1116)* animals on Day 3 of adulthood. Arrows indicate autolysosome-like structures. (D) Quantification of autolysosome-like structures. Data are the mean ± SEM of 14–20 micrographs. *P = 0.0051, Student’s *t*-test. (E) Representative images of overlaid fluorescence and differential interference contrast (DIC) channels showing the intestine of 5-day-old WT and *eat-2(ad1116)* adult animals labeled with LysoTracker Red DND-99. (F). Quantification of fluorescence intensity in the anterior intestine. Data are the mean ± SEM of 10 animals. *P<0.0001, Student’s *t-*test. The experiment was repeated six times with similar results.

During autophagy, LGG-1/ATG8 is sequestered at the membrane of nascent autophagosomes and can be visualized as characteristic punctate structures in animals expressing a GFP-tagged form of LGG-1 [25, 26]. We and others have previously reported that DR (via the *eat-*2 mutation or by dilution of bacterial food source) increases the abundance of GFP::LGG-1–positive punctae in the hypodermal seam cells of L3 larvae and 1-day-old adult animals [5, 7, 8]. To investigate the role of autophagy in the intestine of DR animals, we monitored autophagy by quantifying autophagosomes in the intestine of WT and *eat-2* animals [27]. Unexpectedly, we found that the number of GFP::LGG-1 punctae was significantly lower in the intestine of *eat-2(ad1116)* mutants compared to WT animals on Day 4 of adulthood (**Fig 2A**). This decrease in GFP::LGG-1 punctae was independent of the type of bacteria the animals were fed (**S2A Fig**), and was also observed by confocal microscopy in older Day 7 animals (**S3A Fig**), collectively suggesting that the steady-state levels of autophagosomes in the intestine of dietary-restricted *eat-2* mutants are lower than in WT animals. Since the hypodermis [5, 7] and the intestine (**Figs 2A, S2A and S3A**) of *eat-2* mutants showed a divergent number of autophagosomes, we additionally counted GFP-positive LGG-1 punctae by confocal microscopy in two other tissues, i.e., body-wall muscle and neurons of 3-day-old *eat-2* mutants (**S2B and S2C Fig**). Similar to the hypodermis, the neurons displayed an increased number of autophagosomes while the muscle showed no change compared to WT, highlighting the intestine as the only tissue in which we observed decreased numbers of GFP::LGG-1 punctae. Notably, all *C*. *elegans* longevity models tested to date require autophagy genes for lifespan extension and have increased numbers of LGG-1 punctae in hypodermal cells at the late larval stages compared to wild-type animals [28]. Thus, our intestinal-specific lifespan experiments (**Fig 1**) and GFP::LGG-1 analyses (**Figs 2A, S2 and S3A**) are the first to show a lack of correlation between a requirement for autophagy genes and an increase in LGG-1-positive punctae in long-lived animals.

### The intestine of *eat-2* mutants contains an enlarged acidic compartment and displays accelerated autophagosome turnover

The reduced number of GFP::LGG-1 positive punctae we observed in *eat-2* mutant intestines may be due to a decrease in the formation of autophagosomes, or an increase in the conversion or turnover of autophagosomes. Consistent with the latter hypothesis and in support of increased autophagic activity we observed that *eat-2(ad1116)* mutants had increased mRNA levels of autophagy-related genes (*lgg-1*/*ATG8*, *sqst-1/SQSTM1*) as well as lysosomal genes (*vps-11*, *vha-15*/V-ATPase, *vha-16*/V-ATPase) compared with the levels in WT animals (**Fig 2B**). These results corroborate our previous analysis of *eat-2(ad1116)* mutants [29], which showed increased expression of additional autophagy-related genes important for autophagosome formation (*atg-18/Wipi* and *atg-9*) as well as lysosomal genes (*lmp-1/Lamp1*, *vps-18*, and cathepsins (*cpr-1*, *asp-1*)), and collectively support that *eat-2* mutants have increased autophagy. Based on these observations, we hypothesized that the intestine of *eat-2* mutants might possess an enlarged lysosomal compartment, which could facilitate autophagosome–lysosome fusion and subsequent autophagosome conversion irrespective of a possible concomitant increase in autophagosome formation. To test this, we used transmission electron microscopy to image the intestines of WT and *eat-2(ad1116)* animals to quantify the number of autolysosomal-like structures. Autolysosomes were identified as vacuoles containing contents undergoing degradation [27]. We found that *eat-2(ad1116)* mutants had a significant increase in the number of autolysosomes at Day 3 of adulthood compared to WT (**Fig 2C and 2D**). Similar observations were made in animals at Day 7 of adulthood (**S3B Fig**). Additionally, we found that *eat-2(ad1116*) mutants had increased intestinal staining of acidic organelles by the pH-sensitive fluorophore LysoTracker Red DND-99, a red-fluorescent dye supplemented to media from Day 1 of adulthood and whose fluorescence intensity was quantified on Day 5 (**Fig 2E and 2F**). Collectively, these observations are consistent with an expanded lysosomal compartment in the intestine of *C*. *elegans* during DR.

This increased steady-state level of lysosomes could facilitate an increased conversion rate of autophagosomes, which might be reflected in lower steady-state levels of GFP::LGG-1 positive punctae, as we observed in the intestine of *eat-2* mutants (**Figs 2A, S2A and S3**). In such a scenario, autophagosomal contents would be turned over more frequently. To test this, we blocked autophagy by subjecting WT and *eat-2(ad1116)* animals to RNAi targeting *vha-15*, which encodes a subunit of an essential lysosomal enzyme, V-ATPase [30]. RNAi from the L4 larval stage to Day 4 of adulthood caused a ~4-fold accumulation of GFP::LGG-1 punctae in the intestine of *eat-2(ad1116)* mutants whereas a small, but insignificant ~5% increase was observed in WT animals (**Fig 1A**). Taken together, our results are consistent with the notion that autophagosome conversion and by extension autophagic activity is increased in the intestine of *eat-2* mutants, at least in part facilitated by a larger lysosomal compartment. The data additionally emphasize the importance of employing multiple autophagy assays, including ‘flux’ assays to infer autophagy activity, to complement GFP::LGG-1 reporter assays and thus provide more accurate assessments of the status of autophagy within cells.

### The age-related decline in intestinal barrier function is reduced in *eat-2* mutants

**Fig 3:**
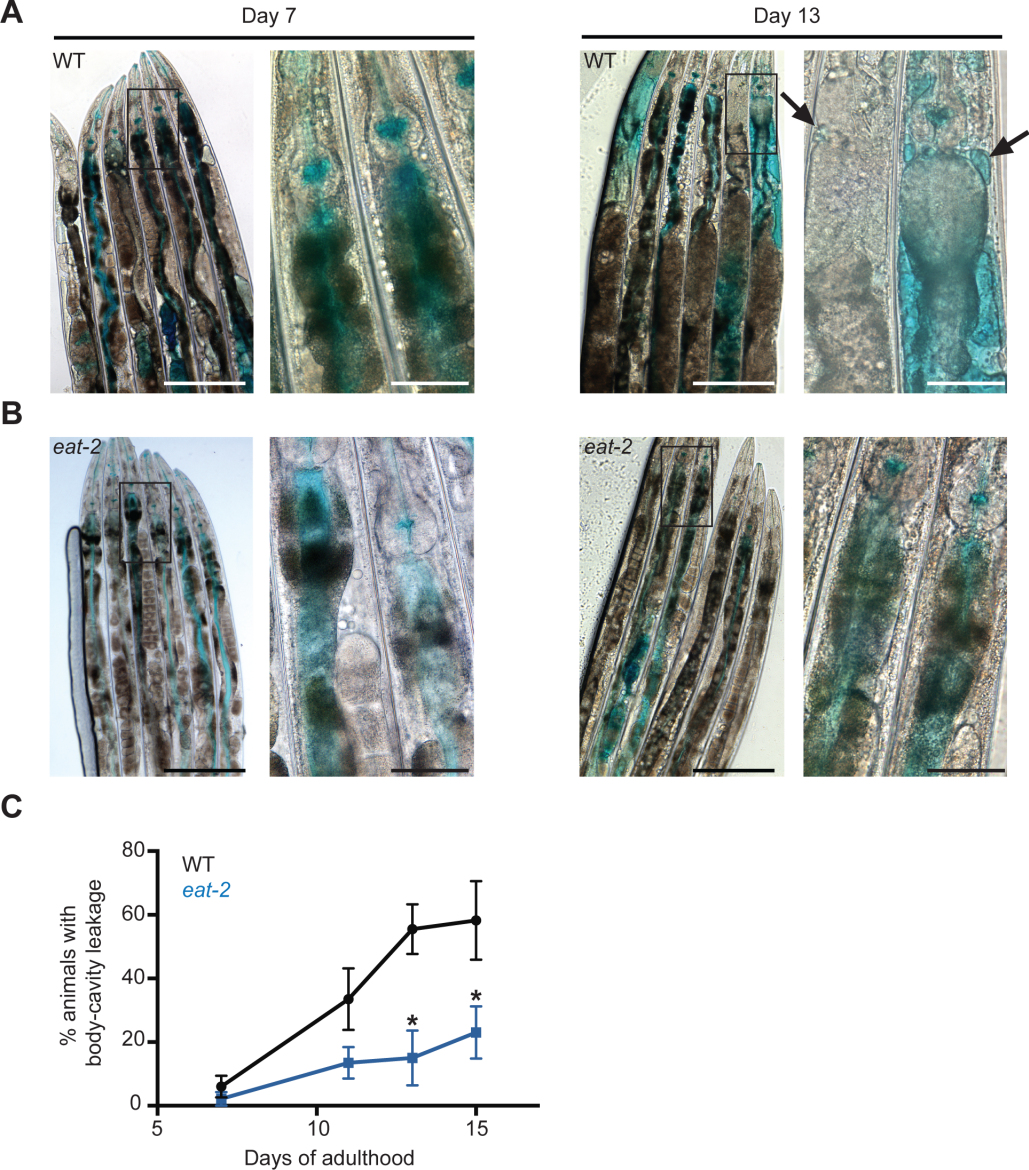
The age-related breakdown of intestinal barrier function is reduced in dietary-restricted *eat-2* mutants. **(A, B)** DIC images of wild-type (WT, N2) animals (**A**) or *eat-2(ad1116)* animals (**B**) after soaking in blue food dye for 3 h on Day 7 (left) and Day 13 (right) of adulthood. Black arrows point to areas where blue dye has leaked from the intestinal lumen into the body cavity, giving rise to the Smurf phenotype. Scale bars, 200 μm (left panels) and 50 μm (right panels, showing higher magnifications of the boxed regions in the left panels). (**C)** Quantification of body-cavity leakage in WT and *eat-2(ad1116)* animals over time. Animals were examined after soaking in dye for 3 h on the indicated days of adulthood (see Methods). Data are the mean ± SEM of three biological repeats per time point, each with 8–10 animals. *P<0.03, Student’s *t-*test.

To further investigate roles of autophagy in the intestine, we next sought to establish an assay that could measure functions of this organ in *C*. *elegans*. To this end, we developed a non-invasive assay to measure *C*. *elegans* intestinal barrier function, as previously done in *Drosophila* [18, 19]. In our protocol, animals were fed for 3 h by submersion in a liquid bacterial culture mixed with a non-absorbable blue food dye, and then visualized microscopically. After incubation, the dye was clearly visible in the intestine of both young and older WT and *eat-2(ad1116)* mutants (**Fig 3A and 3B**). While the dye was contained within the intestine in younger animals, older animals also displayed dye in their body cavity (**Fig 3A and 3B**; as in the *Drosophila* studies, we refer to this as a 'Smurf' phenotype [18, 19]). Specifically, dye leakage was observed around mid-adulthood (Day 7; **Fig 3C**) and increased in frequency with age (~60 percent on Day 15; **Fig 3C**), indicating an age-dependent decline in intestinal integrity similar to observations in *Drosophila* [18, 19]. Notably, the intestinal integrity of *eat-2(ad1116)* mutants was remarkably improved as far fewer *eat-2(ad1116)* than WT animals displayed the Smurf phenotype (~20 percent on Day 15; **Fig 3B and 3C**). These data suggest that the age-related decrease in intestinal integrity in *C*. *elegans* is improved by DR, as observed in *Drosophila* [19, 20]. We also tested *daf-2(e1370)* insulin/IGF-1 receptor mutants, another long-lived mutant [31] in the Smurf assay. Interestingly, these mutants maintained their intestinal integrity remarkably well over time (only ~5 percent on Day 16; **S4A Fig**), suggesting a broader correlation between longer lifespan and improved intestinal integrity.

### Autophagy genes act cell autonomously to improve intestinal integrity in dietary-restricted *eat-2* mutants

**Fig 4:**
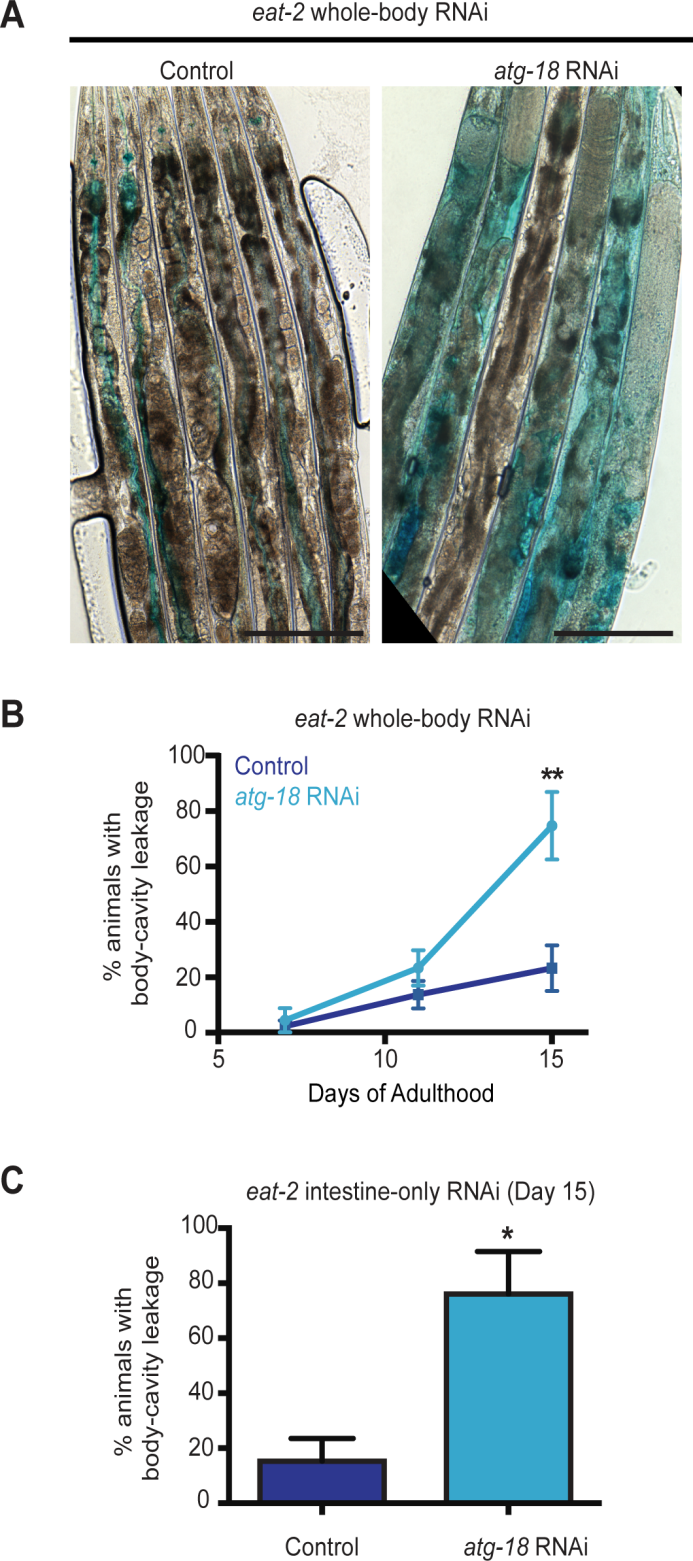
Autophagy-gene knockdown reduces the intestinal integrity of dietary-restricted *eat-2* mutants. **(A)** DIC images of *eat-2(ad1116)* animals fed from Day 1 of adulthood with bacteria containing empty vector (control) or expressing *atg-18/Wipi*-targeting dsRNA, and then soaked in blue food dye for 3 h on Day 15 of adulthood. Scale bars, 200 μm. (**B**) Quantification of body-cavity leakage in *eat-2(ad1116)* animals fed from Day 1 of adulthood with bacteria containing empty vector (control) or expressing *atg-18/Wipi*-targeting dsRNA. Data are the mean ± SEM of three biological repeats, each with 8–10 animals per time point. **P = 0.005, Student’s *t*-test. (**C**) Quantification of body-cavity leakage in 15-day-old *eat-2(ad1116)*; *rde-1(ne219); nhx-2p*::*rde-1* transgenic animals fed from Day 1 of adulthood with bacteria containing empty vector (control) or expressing *atg-18/Wipi*-targeting dsRNA. Data are the mean ± SEM of three biological repeats, each with 8–10 animals. *P = 0.025, Student’s *t*-test.

Based on our observations that *eat-2* mutants display improved intestinal barrier function and enhanced autophagosomal turnover in the intestine compared with WT animals, we next asked whether autophagy was required to maintain the intestinal integrity of these mutants. WT and *eat-2* mutants were subjected to whole-body *atg-18/Wipi* RNAi during adulthood, and the dye-leakage assay was performed on Days 7, 11, and 15 of adulthood. More *eat-2(ad1116)* mutants with reduced *atg-18/Wipi* levels displayed the Smurf phenotype by Day 11 and furthermore at Day 15 (**Fig 4A and 4B**) than control animals, largely coinciding with the time at which *eat-2(ad1116)* animals subjected to *atg-18/Wipi* RNAi would start to be at high risk of dying (**Fig 1 and S1 Table**). In contrast, *atg-18/Wipi* RNAi had no significant effect on dye leakage in WT animals at any time point measured (**S4B Fig**). Similar trends were observed in *eat-2(ad1116)* mutants subjected to whole-body *vha-15*/V-ATPase RNAi (**S4C Fig**) or *lgg-1*/*lgg-2/ATG8* RNAi (**S4D Fig**) at Day 15 of adulthood. These data indicate that whole-body RNAi of autophagy genes reduced the protective effect of the *eat-2* mutation on intestinal barrier function just like inhibition of autophagy prevents the long lifespan of *eat-2* mutants, yet cause relatively small or non-significant effects on lifespan or intestinal barrier function in WT animals [5, 6] (**S1 Table**).

To determine whether autophagy specifically within the intestine contributes to the barrier function, we subjected *eat-2; rde-1* mutants expressing *rde-1* from the *nhx-2* promoter to *atg-18/Wipi* RNAi during adulthood and performed the dye-leakage assay on Day 15. We found that intestine-specific *atg-18/Wipi* RNAi was sufficient to significantly increase intestinal leakage in *eat-2(ad1116)* mutants compared to control-treated animals (**Fig 4C**). These findings indicate that autophagy can act cell autonomously in the intestine of *eat-2* mutants to maintain barrier function, a conserved healthspan parameter that can be experimentally measured in *C*. *elegans*.

### Autophagy genes act cell non-autonomously to affect the motility of *eat-2* mutants

**Fig 5:**
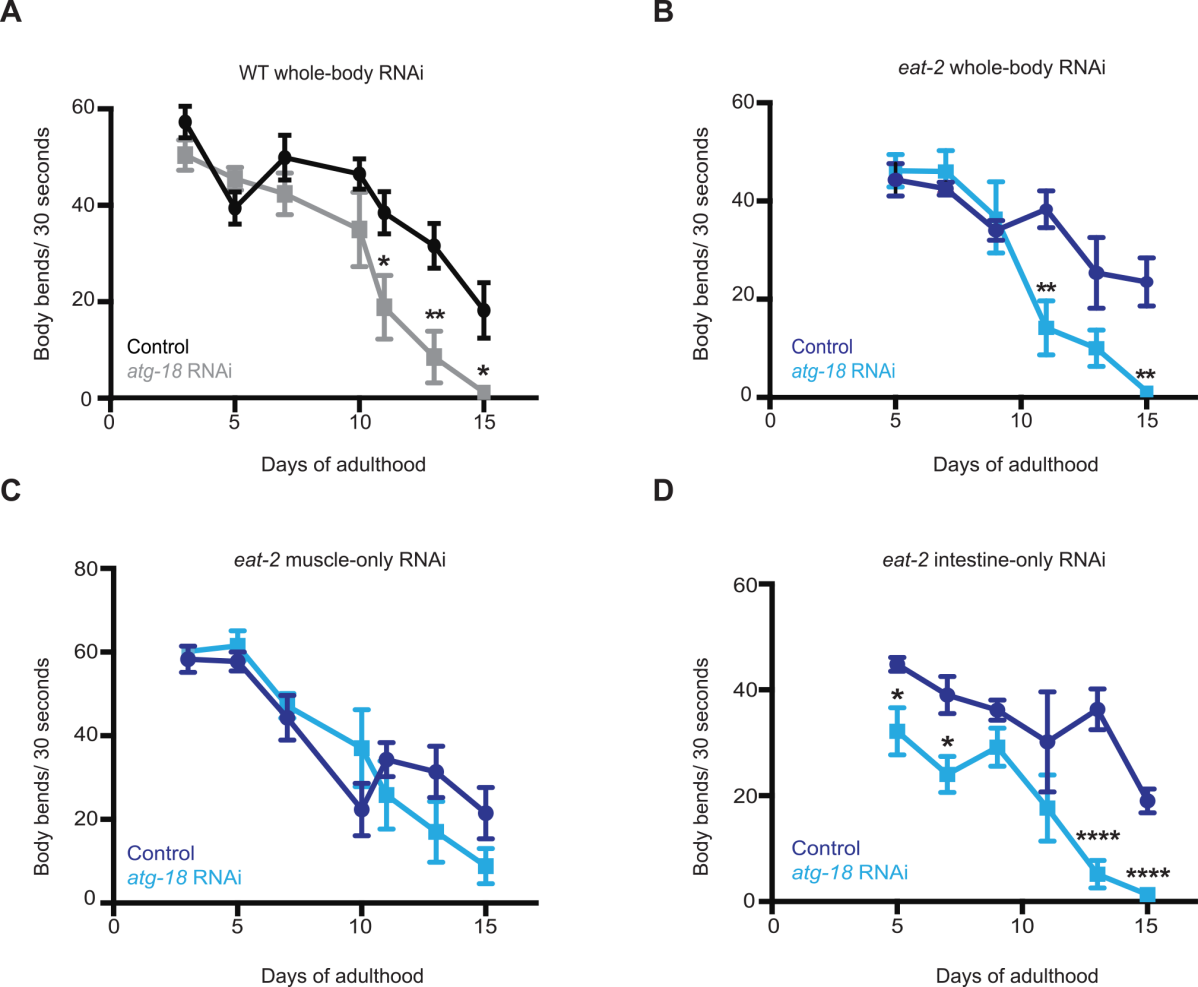
Autophagy gene knockdown decreases the motility of wild-type and dietary-restricted *eat-2* animals. Quantification of body bends in wild-type (WT, N2) animals (**A**) and *eat-2(ad1116)* mutants (**B**) fed from Day 1 of adulthood with bacteria containing empty vector (control) or expressing *atg-18/Wipi*-targeting dsRNA. Data are the mean ± SEM of 6 animals per time point. For WT animals, *P = 0.03 on Day 11, **P = 0.008 on Day 13, and *P = 0.014 on Day 15, Student’s *t*-test. For *eat-2(ad1116)* animals, **P = 0.004 on Day 11 and **P = 0.002 on Day 15, Student’s *t*-test. The experiments were repeated three times with WT animals or eleven times with *eat-2(ad1116)* with similar results. (**C, D**) Quantification of body bends in *eat-2(ad1116)*; *sid-1(qt9); myo-3p*::*sid-1* (**C**) and *eat-2(ad1116)*; *rde-1(ne219); nhx-2p*::*rde-1* (**D**) transgenic animals fed from Day 1 of adulthood with bacteria containing empty vector (control) or expressing *atg-18/Wipi*-targeting dsRNA. Data are the mean ± SEM of 10–12 animals per time point. For (**C**), the differences were insignificant by Student’s *t*-test on all days; however, whole-body RNAi controls were carried out in parallel and showed effects, confirming RNAi clone efficiency. For (**D**), *P = 0.021 on Day 5, *P = 0.012 on Day 7, ****P<0.0001 on Day 13 and 15, Student’s *t*-test. Similar motility phenotypes were observed in animals subjected to the same RNAi treatments on solid media plates. Experiments were repeated three times with similar results.

Additional healthspan assays can be used to assess physiological functions in *C*. *elegans* [32]. To more broadly explore roles for autophagy in *C*. *elegans*, we investigated the effects of autophagy on motility. This activity engages muscular and neuromuscular functions of the animal and is a common measure of *C*. *elegans* healthspan [33–35]. To quantify motility, we counted body bends in WT and *eat-2* animals swimming freely in liquid media. While Day 3 *eat-2(ad1116)* animals were slightly more active than WT animals, the motility of older *eat-2(ad1116)* mutants did not significantly differ from that of WT animals (**S5 Fig**), as previously reported for *eat-2(ad1113)* mutants [34]. Interestingly, whole-body RNAi of *atg-18/Wipi* caused a dramatic decline in motility that reached statistical significance on Day 11 in both WT (**Fig 5A**) and *eat-2(ad1116)* animals (**Fig 5B**). Motility defects generally appeared prior to body-cavity leakage, which first became highly significant on Day 15 in *eat-2(ad1116)* mutants subjected to whole-body autophagy gene RNAi (**Fig 4B**). Thus, motility decline induced by whole-body autophagy gene knockdown in *eat-2* mutants may therefore not be linked to lifespan or to intestinal leakage. Consistent with this notion, WT animals subjected to whole-body *atg-18/Wipi* RNAi displayed decreased motility (**Fig 5A**), but had the same degree of intestinal leakage at least up to Day 15 (**S4B Fig)** as control animals.

The decline in motility caused by whole-body autophagy gene knockdown might reflect changes in neuromuscular functions. To specifically investigate the contribution of muscle, we introduced a myosin MYO-3::GFP reporter highlighting muscle fibers into *eat-2(ad1116)* mutants and subjected these transgenic animals as well as WT animals expressing MYO-3::GFP to whole-body *atg-18/Wpi* and control RNAi. While Day 15 WT animals experienced a significant sarcomere deterioration in response to *atg-18/Wipi* RNAi compared to control treatments, we observed no obvious differences in the appearance of body-wall muscle fibers in Day 15 *eat-2(ad1116)* mutants subjected to control or whole-body *atg-18/Wipi* RNAi (**S6A and S6B Fig**) despite *atg-18/Wipi* RNAi-treated *eat-2* animals being clearly movement impaired (Unc). These data indicate that different mechanisms may underlie the motility decline observed in WT animals compared to dietary-restricted *eat-2* animals following autophagy inhibition. To further address the role of autophagy in the muscle in *eat-2* mutants, we created an *eat-2(ad1116); sid-1(qt9)* double mutant expressing *sid-1* from the *myo-3* promoter to be able to inhibit autophagy specifically in the muscle (**S1A Fig**). Muscle-specific knockdown of *atg-18/Wipi* in adult *eat-2(ad1116)* animals however caused only minor effects on motility (**Fig 5C**). In contrast, muscle-specific *atg-18/Wipi* RNAi significantly shortened the long lifespan of these animals (**S7 Fig and S1 Table**). These observations indicate that the body-wall muscle in *eat-2; sid-1; myo-3p*::*sid-1* transgenic animals indeed possesses RNAi processing ability **(S1A Fig),** and that autophagy in the body-wall muscle of *eat-2* mutants is critical for longevity, as is the case in WT mice [36]. We similarly tried to address functions for autophagy in the neurons of *eat-2* mutants by making an *eat-2(ad1116); sid-1(qt9)* double mutant expressing *sid-1* from the neuronal *rab-3* promoter. While we observed that *atg-18/Wipi* RNAi significantly shortened the long lifespan of this strain (**S1 Table**), the *rab-3* promoter appeared leaky to the intestine starting around Day 5 of adulthood (**S1A and S1B Fig**, see also Methods). Therefore we were not able to conclusively determine the neuronal effects of autophagy at this point.

While autophagy in the body-wall muscle of *eat-2* mutants may contribute to their longevity, the motility defects observed after whole-body, but not muscle-specific, disruption of autophagy in *eat-2* mutants may originate as a consequence of autophagy disruption in tissues other than the body-wall muscle. Interestingly, we found that *eat-2(ad1116); rde-1(ne219)* mutants expressing *nhx-2p*::*rde-1* subjected to intestinal-specific *atg-18/WWipi* RNAi displayed significantly reduced motility compared with animals subjected to control RNAi, even relatively early in adulthood (**Fig 5D**). These observations indicate that autophagy in the intestine of dietary-restricted animals has physiological effects on distal tissue function. As such, our data indicate that autophagy inhibition in the intestine during DR not only accelerates a cell-autonomous function (i.e., decline in intestinal barrier function) but also has cell non-autonomous consequences in reducing motility.

## Discussion

Here we show a critical role for autophagy in the intestine to ensure lifespan extension of dietary-restricted *eat-2* mutants. Moreover, autophagy inhibition in this major tissue accelerated an age-dependent decline in the intestinal barrier function and in motility of dietary-restricted animals, thus highlighting both cell autonomous and non-autonomous consequences of inhibiting autophagy in a single tissue. Since we found that dietary-restricted animals display an enlarged lysosomal compartment and possibly increased autophagosome conversion in the intestine, we propose that increased autophagic activity in this tissue extends the lifespan of dietary-restricted animals by improving multiple aspects of organismal fitness.

We used several assays to monitor autophagy in dietary-restricted *eat-2* animals, including a widely used GFP::LGG-1 reporter that reflects steady-state levels of autophagosomes [25, 26]. Its subcellular localization into GFP-positive punctae is increased in the hypodermis of larvae of all long-lived *C*. *elegans* mutants investigated so far, including *eat-2(ad1116)* mutants [5, 7, 8]. We observed GFP::LGG-1 punctae in adult WT and *eat-2* animals in all tissues inspected, i.e., intestine, muscle and neurons, consistent with a recent report using an enzymatic assay to show that these major tissues can modulate autophagy [37]. Notably, of the three tissues analyzed here the intestine stood out as the only tissue with a decrease in GFP::LGG-1 counts in *eat-2* animals compared to WT. We used additional steady-state analyses as well as a ‘flux’ assay that collectively indicated that the intestine of *eat-2* mutants possesses increased autophagosome turnover. Together, these intestinal experiments represent the most comprehensive analysis to date of the various steps in the autophagy process in a tissue of *C*. *elegans*, and highlight the importance of using multiple assays to monitor the autophagy process [3]. While neurons showed an increase and body-wall muscle showed no difference in the number of GFP::LGG-1 punctae in 3-day-old adult *eat-2* mutants compared to WT animals, additional assays are needed to conclusively evaluate the status of autophagy in these tissues. One interpretation of our intestinal data is that autophagosome conversion is more rapid in the intestine of dietary-restricted *eat-2* mutants than in WT animals, possibly facilitated by the enlarged lysosomal compartment observed in dietary-restricted mutants. Additional biochemical experiments will be needed to measure the kinetics of the overall autophagy process and its individual steps in WT and dietary-restricted animals to directly address this possibility.

We found that inhibition of several autophagy genes (i.e., *atg-18/Wipi* and *lgg-1/lgg-2/ATG8)* in the intestine of *eat-2* animals was sufficient to significantly shorten the lifespan, as previously observed following whole-body knockdown of these (this study) and additional autophagy genes (i.e., *unc-51/ATG1/Ulk1*, *bec-1/ATG6/Beclin1*, *vps-34* and *atg-7*) [5, 6, 8]. These observations suggest that intestinal autophagy contributes to DR-mediated lifespan in *C*. *elegans*. To investigate the possible functions of intestinal autophagy, we developed a novel dye-leakage assay (termed the ‘Smurf’ assay) that allowed us to quantify the intestinal barrier function in *C*. *elegans*, as has previously been done in *Drosophila* [18, 19]. (We note that, while this manuscript was under revision, Michael Rera and his collaborators independently reported a dye-leakage assay in *C*. *elegans*, as well as in zebrafish [38]). Our results indicate that *eat-2(ad1116)* animals are more resistant than WT animals to the age-associated increase in dye leakage, similar to observations in flies [19, 20], a scenario that appears to extend to additional long-lived mutants, since *daf-2* insulin/IGF-1 receptor mutants were remarkably resistant to dye leakage. While we cannot rule out a potential contribution from hypodermal leakage in our assay (since the animals are soaked in dye), our findings are consistent with a previous study of WT *C*. *elegans* that used several cytological methods to demonstrate an age-dependent decline in intestinal integrity, associated with irregular lumenal shape, changes in microvilli, and loss of nuclei [39]. Interestingly, whole-body autophagy gene inhibition during adulthood accelerated the breakdown of intestinal integrity in dietary-restricted *eat-2* animals but had no significant effect in WT animals; this is similar to the lifespan-extending effects of autophagy inhibition in *eat-2* mutants, but generally with small or no significant effect in WT animals [5, 6]. This finding implies that increased levels of autophagy in the intestine is required for the improved intestinal barrier function in dietary-restricted animals, and could underlie the longevity of these animals. Indeed, the decline in intestinal integrity of *eat-2* animals generally coincided with the decrease in survival observed at Day 15 in these animals. However, this observation does not exclude the possibility that basal autophagy could be important for the intestinal barrier function in WT animals. Our finding that autophagy inhibition within the intestine of *eat-2* mutants is sufficient to accelerate the decline in intestinal integrity suggests that autophagy normally functions cell autonomously to support intestinal barrier function in dietary-restricted animals. This is consistent with observations in *Drosophila*, where increased expression of *Atg1/Ulk1* induces autophagy and improves the intestinal barrier function [40]. The barrier function itself is maintained via protein-protein networks that form adhesive complexes of desmosomes, adherens junctions, and tight junctions [41]. Notably, a recent study suggested a role for autophagy in turnover of the pore-forming tight junction protein Claudin-2 in nutrient-starved human intestinal epithelial cells, leading to less permeable junctions [42]. Since our findings suggest a link between beneficial autophagic turnover and improved intestinal barrier function in *eat-2* mutants, it would be interesting to investigate autophagy-dependent maintenance of tight junctions in dietary-restricted animals.

We also assessed the relationship between autophagy and motility, an important healthspan parameter that is known to decline with age, and found that autophagy knockdown significantly reduced the motility of both WT and *eat-2* animals. This observation is consistent with reduced climbing activity in adult *Atg7* mutant flies [43]. Since autophagy gene RNAi significantly shortens the lifespan of *eat-2* mutants but generally has relatively small or non-significant effects in WT animals, these findings uncouple the motility decline from lifespan shortening and suggest that animals with reduced autophagy levels may not die simply as a consequence of decreased motility. Autophagy could influence *C*. *elegans* motility in a number of ways, including via autonomous effects in muscle cells. In this regard, inhibition of autophagy in skeletal muscle cells of WT mice reduces lifespan, exacerbates mitochondrial dysfunction, and significantly impairs both muscle strength and neuromuscular synaptic function [9, 10, 36]. We observed similar effects on sarcomere integrity in WT *C*. *elegans* subjected to autophagy gene inhibition, whereas the muscle fibers in *eat-2* mutants with impaired autophagy appeared similar to the control. Moreover, we found that muscle-specific autophagy inhibition had no significant effect on the motility of *eat-2* animals, while the same treatment was sufficient to prevent lifespan extension in *eat-2* mutants. These observations suggest a longevity role for autophagy not only in the intestine but also in body-wall muscle, as suggested in WT mice [36], and indicate differences between the role of basal autophagy versus autophagy induced by DR in maintaining muscle integrity that are still to be elucidated.

Interestingly, we observed that intestinal autophagy was required for the motility of *eat-2* mutants, highlighting a cell non-autonomous function of autophagy in the intestine. One explanation for this observation is that intestinal inhibition of autophagy may cause deterioration of this organ, as implied by our Smurf assays. In turn, this could have deleterious effects on distal tissues such as muscle or motor neurons by reducing nutrient availability or other signals from the intestine. However, *eat-2* animals subjected to whole-body autophagy RNAi showed significant motility defects on Day 11, before significant intestinal barrier dysfunction was detected on Day 15. These observations therefore indicate that intestinal dysfunction (as measured by the Smurf assay) may not be causally related to the motility decline observed upon whole-body autophagy gene knockdown. Rather other potential functions of autophagy in the intestine (e.g., turnover of intestinal nuclei [39], or of mitochondria [44]) may be important for the integrity of this tissue. As noted above, inhibition of intestinal autophagy in *eat-2* mutants could affect motility because metabolic and/or endocrine functions of the intestine could be impaired. Such metabolic/endocrine functions of the intestine may be particularly important in dietary-restricted animals in which nutrient availability is already limited. Alternatively, intestinal inhibition of autophagy in *eat-2* mutants could impair the detection/receipt of signals from distal, non-intestinal tissues that are important for maintaining intestinal function. To this point, we created *eat-2*; *sid-1* animals expressing *sid-1* from the neuronal *rab-3* promoter, and we observed that autophagy knockdown in this strain significantly shortened the long lifespan of these animals (**S1 Table** and **S1A Fig**). While this observation indicates a possible longevity role for autophagy in neurons, as in the intestine and body-wall muscle, we found the *rab-3* promoter in addition to neurons also drove expression in the intestine in older animals, thus compromising the interpretation of our lifespan data. Further studies will be needed to elucidate the interaction between autophagy in the intestine and other tissues, and to determine how they affect intestinal integrity, motility, and longevity.

In conclusion, our study provides insights into the contribution of intestinal autophagy and the importance of inter-tissue communication in the healthspan of dietary-restricted *C*. *elegans*. Further work to identify the cellular and molecular pathways that sense DR, regulate specific steps of the autophagy process, and mediate inter-tissue signaling will be important to gain a more complete understanding of the homeostatic role of autophagy in maintaining the health and longevity of an organism.

## Materials and Methods

### Strains

Strains were maintained and cultured under standard conditions at 20°C using *E*. *coli* OP50 as a food source [45]. For RNAi experiments, animals were grown on HT115 bacteria transformed with empty vector or plasmid encoding the appropriate gene-specific dsRNA (see below). See **S2 Table** for strains created for and used in this study.

RNAi efficiency was assessed in all *eat-2* tissue-specific RNAi strains with RNAi clones encoding genes expressed in specific tissues (**S1A Fig**), as we have done previously [46, 47]. As expected, neither *eat-2(ad1116); rde-1(ne219)* nor *eat-2(ad1116); sid-1(qt9)* double mutants showed any phenotypes in these tests. We also tested RNAi efficiency in *eat-2(ad1116); sid-1(qt9)* strains expressing *sid-1* from different promoters with a *tdTomato* RNAi clone against the fluorescent co-injection marker (**S1A Fig**). In these analyses, the newly made tissue-specific *eat-2; sid-1* transgenic strains all showed the expected processing (**S1A Fig**). However, the *eat-2; sid-1; rab-3p*::*sid-1* neuronal RNAi strain also showed signs of intestinal RNAi-processing capabilities, i.e., on *elt-2* RNAi. Consistent with this observation, we found that transgenic animals expressing a *rab-3p*::*mCherry* transcriptional reporter started showing expression in the intestine around Day 5 (**S1B Fig**). Collectively, these observations argue that the *rab-3* promoter is leaking to the intestine. In our RNAi analyses, we also observed that *bli-4* and *bli-3* RNAi produced phenotypes in *eat-2; sid-1; gly-19p*::*sid-1* mutants (**S1A Fig**). While this is consistent with the *gly-19* promoter possibly expressing in the hypodermis, in addition to the intestine, we did not observe visible ectopic expression of the *gly-19p*::*tdTomato* co-injection marker in *eat-2; sid-1* animals up to 10 days of age. However, we have occasionally observed old WT animals carrying the same array display ectopic expression to head neurons.

In a different line of experiments, we observed GFP knockdown in the intestine of *eat-2; sid-1* double mutants crossed to a translational *lgg-1p*::*gfp*::*lgg-1* reporter when subjected to *gfp* RNAi. Although this observation suggests that some degree of RNAi processing may take place in the intestine of all strains, this did not appear to contribute to the changes in lifespan, body-cavity leakage or motility we observed in *eat-2; sid-1* transgenic strains subjected to tissue-specific autophagy gene RNAi, since *eat-2; rde-1* or *eat-2; sid-1* double mutants did not show significant phenotypes in these assays when subjected to autophagy RNAi.

### Construction of transgenic strains

For the *rgef-1*:*gfp*::*lgg-1* construct, PCR products were generated from the *C*. *elegans rgef-1*/F25B3.3 cDNA using primers: Fwd 5′ GGG GAC AAG TTT GTA CAA AAA AGC AGG CTG GGC ATG CTA AGT GAT CTG ACC TCG CGC CCC 3′ and Rvs 5′ GGG GAC CAC TTT GTA CAA GAA AGC TGG GTG GGT ACC GTC GAT GCC GTC TTC 3′ (1.9 kb). The *rgef-1* promoter-containing plasmid was a gift from Dr. Andrew Dillin. The PCR products were sequenced and introduced into the original *lgg-1p*::*gfp*::*lgg-1* plasmid [25] to generate pMH882, which was injected at 20 ng/μl, with 34 ng/μl of *unc-122p*::*rfp* as a co-injection marker [48], into wild-type animals and integrated by γ-irradiation followed by outcrossing to WT (N2) animals.

To construct plasmids expressing *sid-1* cDNA driven by various promoters, full-length *sid-1* cDNA (2330 bp) was cloned from first-strand worm cDNA by PCR amplification and inserted in the *C*. *elegans* expression vector pPD95.77 using *Xma I* and *Age* I restriction enzymes. The *unc-54* 3’ UTR was PCR amplified with a 5’ *Age I* site and a 3’ *BsiW I* site and cloned into the *sid-1*/pPD95.77 vector in the place of the *gfp*::*unc-54* 3’ UTR fragment. To create the intestinal expression construct, the *gly-19* promoter was cloned in front of the *sid-1* cDNA using *Sph I* and *Xma I* enzymes. As co-injection marker for the intestinal expression vector, *tdTomato* was amplified from the original *tdTomato* sequence (in pCMV/*tdTomato*, a gift from Dr. Roger Tsien) and cloned into the *C*. *elegans* expression vector pPD95.77 using *Age I* and *Bsm I* (s260), followed by cloning in the *gly-19p* promoter sequence using *Sph I* and *Xma I*. The *sid-1*-containing vector, and the *tdTomato-*containing co-injection marker were microinjected into the gonads of adult *eat-2(ad1116); sid-1(qt9)* hermaphrodite animals at a concentration of 10 ng/μl. The DNA mix for injection was brought to a final total concentration of 100 ng/μl using pPD61.125 as “Filler” DNA. Integration was performed by γ-irradiation followed by outcrossing to *eat-2(ad1116); sid-1(qt9)* animals.

Plasmids expressing *mCherry* cDNA driven by the *rab-3* promoter were purchased from Addgene and injected (15 ng/μg) into WT (N2) animals along with 100 μg/μg of pRF4/*rol-6* marker.

### RNAi clones and methods

HT115 bacteria carrying empty vector (plasmid L4440) were used as RNAi controls. The following RNAi clones were obtained from the Ahringer library (JA) or the Vidal RNAi library (MV): *atg-18/F41E6*.*13* (MV), *lgg-1/C32D5*.*9* (JA), *lgg-2/ZK593*.*6* (JA). Construction of *tdTomato* RNAi clone: *tdTomato* was amplified from vector s260 and cloned into plasmid L4440. All RNAi clones were verified by sequencing. We note an oversight in our earlier report that *unc-51*/*Y60A3A*.*1*, *atg-7/M7*.*5*, and *atg-18* RNAi failed to significantly reduce lifespan in *eat-2* mutants [5]: *atg-18* RNAi was erroneously included in this list.

For RNAi experiments, HT115 bacteria were grown in liquid LB medium containing 0.1 mg/ml carbenicillin (BioPioneer), and then 80 μl samples were spotted onto 6 cm NGM plates supplemented with carbenicillin and allowed to grow for 1–2 days at room temperature. dsRNA expression was induced by addition of 80 μl 0.1 M IPTG (Promega) to the bacterial lawn, and eggs or adult worms were then transferred to the plates. For whole-life RNAi, animals were synchronized by hypochlorous acid treatment and the eggs were placed on NGM plates seeded with dsRNA-expressing bacteria. For adult-only RNAi, animals were synchronized by hypochlorous acid treatment, eggs were placed on NGM plates seeded with OP50 bacteria and allowed to hatch, and worms were then transferred to NGM plates seeded with dsRNA-expressing bacteria on Day 1 of adulthood.

### Lifespan analyses

Lifespan was measured at 20°C as previously described [49]. In brief, ~100 synchronized animals were examined every second day of adulthood and were scored as dead if they failed to respond to gentle prodding with a platinum wire pick. Integrated, outcrossed strains were used for tissue-specific RNAi experiments. Animals were censored from the analysis if they became desiccated on the edge of the plate, escaped, ruptured, or suffered from internal hatching. Statistical analysis using the log-rank (Mantel-Cox) method was performed with Stata software (StataCorp). We used multiple versions of *eat-2(ad1116)* mutant strains (i.e., CF1908, MAH95, and MAH458; **S2 Table**), which were regularly outcrossed due to occasional increases in male progeny or sterility in the population.

We note that multiple methods of DR have been shown to increase lifespan in *C*. *elegans* [50]. While we have shown that several of these paradigms cause changes in the GFP::LGG-1 reporter [5], it remains possible that the tissue-specific role and regulation of autophagy in animals subjected to DR in different ways may differ.

### GFP::LGG-1 autophagy reporter measurements

For tissue-specific analyses, WT or mutant animals expressing GFP::LGG-1 in the muscle and intestine (from the *lgg-1* promoter, DA2123 [51]) or in neurons (from the *rgef-1* promoter, see above) were raised at 20°C, and GFP::LGG-1 punctae were visualized and counted on Day 3 of adulthood. Day 3 was chosen for our autophagy analyses since this was the first time point in motility tests. Animals were mounted on a 2% agarose pad in M9 medium containing 0.1% NaN_3_, and Z-stack images were taken at 0.6 μm intervals using an LSM Zeiss 710 scanning confocal microscope at 630× magnification. GFP::LGG-1 punctae were quantified as follows: for the muscle, punctae in one 1000 μm^2^ area per 0.6 μm slice per animal; for the neurons, total punctae between the pharyngeal bulbs in one 0.6 μm slice per animal; for the intestine, punctae in one proximal cell (with visible nucleus) per 0.6 μm slice per animal.

For *vha15*/V-ATPase RNAi experiments, animals expressing GFP::LGG-1 from the endogenous reporter were raised from L4 larval stage to Day 4 of adulthood on bacteria containing empty vector or expressing *vha-15*/T14F9.1 dsRNA (JA). Animals were mounted as described above and GFP::LGG-1 punctae were quantified using Zeiss Imager Z1 fluorescence microscope, as previously described [27]. GFP::LGG-1 punctae were scored in the proximal region of the intestine, encompassing the first three pairs of intestinal cells starting beyond the pharyngeal grinder.

### Transmission electron microscopy

Animals were raised at 20°C, collected on Day 3 of adulthood, and prepared for analysis as described [27]. Grids were viewed using a Philips CM10 electron microscope (FEI) equipped with a Morada digital camera (Olympus) and iTEM software (Olympus SIS). Autolysosomes were identified as vesicles containing material undergoing degradation in 5000-fold magnified micrographs of a cross-section of the intestine. Statistical analysis was performed using GraphPad Prism.

### LysoTracker staining

LysoTracker Red DND-99 (Invitrogen) was added at 2 μM to NGM medium before plates were poured. Animals were raised at 20°C, placed on LysoTracker-supplemented plates on Day 1 of adulthood, and examined on Day 5. Animals were mounted on a 2% agarose pad in M9 medium containing 0.1% NaN_3_ and analyzed using a Zeiss Imager Z1 fluorescence microscope. Image analysis was performed by selecting a 51.3 × 51.3 pixel area of the anterior intestine and measuring the integrated intensity of LysoTracker fluorescence in images taken after a 100 ms exposure (ImageJ software; National Institutes of Health). Statistical analysis was performed using GraphPad Prism on data acquired from ~10 animals per condition.

### Quantitative RT-PCR

Total RNA was isolated from an age-synchronized population of ~2000 animals flash frozen in liquid nitrogen on Days 1 or 5 of adulthood. RNA was extracted with TRIzol (Life Technologies) and purified using a Qiagen RNeasy kit with an additional DNA digestion step performed with a Qiagen DNase I kit. M-MuLV reverse transcriptase and random 9-mer primers (New England Biolabs) were used for reverse transcription of 1 μg of RNA per sample [52]. Quantitative PCR was performed using SYBR Green Master Mix and a Roche LC480 LightCycler. A standard curve was included for each primer using serial dilutions of a mixture of cDNAs, and the observed CT values were converted to relative values according to the standard curve obtained with the relevant primers. Target gene mRNA levels were normalized against the geometric mean mRNA levels of the housekeeping genes *ama-1* (large subunit of RNA polymerase II) and *nhr-23* (nuclear hormone receptor) [29, 53]. Primer sequences can be found in **S3 Table.** Each biological sample was analyzed with three technical replicates. The mean ± SEM for each mRNA was calculated and the data were analyzed by one-way ANOVA using GraphPad Prism.

### Intestinal barrier function assay

Animals were raised as described above for lifespan assays. On specific days, ~10 animals were removed from the NGM plates and suspended for 3 h in liquid cultures of standard OP50 bacteria (grown overnight) mixed with blue food dye (Spectrum FD&C Blue #1 PD110, 5.0% wt/vol in water). We detected no reduction in the rate of dye uptake in *eat-2* mutants, despite their reduced pumping rate [21]. Animals were then transferred to NGM plates seeded with OP50 bacteria and analyzed for the presence or absence of blue food dye in the body cavity using a Leica DFC310 FX microscope under 40x magnification. For each time point, three or more independent experiments were carried out, each with 8–10 animals per strain and/or treatment. Data were analyzed using GraphPad Prism.

We often observed an age-related increase in the frequency of WT and *eat-2* mutant worms with blue food dye in the germline, increasing from ~10% of worms on Day 7 to ~50% on Day 15. It is unclear whether the dye entered the germline through the body cavity or the vulva; however, many animals had dye in the germline but not the body cavity, suggesting entry through the vulva. For this reason, we included in the analysis animals with dye present in both the germline and body cavity, and excluded animals with dye present only in the germline. Multiple versions of outcrossed *eat-2(ad1116)* mutants were used for this assay (see Lifespan analysis section).

### Motility assay

Animals were raised as in lifespan assays. On specific days, animals were removed from NGM plates, suspended individually in M9 medium, and the number of body bends in a 30 s period was counted. One body bend was counted every time the part of the worm just behind the pharynx reached a maximum bend in the opposite direction from the bend last counted. Data were analyzed using GraphPad Prism. Multiple versions of outcrossed *eat-2(ad1116)* mutants were used for this assay (see Lifespan analysis section).

### Sarcomere analysis

Animals were raised at 20°C, moved to RNAi plates on Day 1 and analyzed on Day 15. Animals were mounted on a 2% agarose pad in M9 medium containing 0.1% NaN_3_ and analyzed using a Zeiss Imager Z1 fluorescence microscope. Image analysis was done manually similar to a previous study [54], and we scored for fragmentation, irregular orientation or broken MYO-3::GFP-positive structures. The degree of these phenotypes was scored on a scale between 1–3 (1 least severe, 3 most severe), and the sum of all scored events were averaged. The analysis was repeated by another person and found to give similar results. Statistical analysis was performed using GraphPad Prism on data acquired from 6–10 animals per condition.

## Supporting Information

S1 Fig RNAi tests of main strains used in this study, and analysis of *rab-3* promoter in aged animals. (**A**) Main strains were tested on RNAi clones encoding genes expressed in major tissue types in *C*. *elegans*, i.e., *elt-2* (intestine), *bli-4* (hypodermis and intestine), *bli-3* (hypodermis), and *unc-112* (muscle). These RNAi clones all gave strong phenotypes in *eat-2(ad1116)* single mutants, but showed no phenotype in RNAi-deficient *eat-2(ad1116); rde-1(ne219)* and *eat-2(ad1116); sid-1(qt9)* double mutants. +, strain produced a similar phenotype as seen in *eat-2* single mutants. -, strain did not produce an obvious phenotype. *A *tdTomato* RNAi clone was also used to assess RNAi efficiency in tissue-specific *sid-1* RNAi strains (by means of the *tdTomato* co-injection marker used in the construction of these strains). The percentages of reduction between control and *tdTomato* RNAi observed were AGD803, -85%, *P*<0.0001; AGD631, -20%, *P*<0.0001; AGD973, -16%, *P*<0.01, Student’s *t*-test. Similar results were observed in at least one other repeat. (**B**) *rab-3*::*mCherry* expression was assessed in wild-type animals at different ages. The strain (MAH338) showed age-dependent, ectopic mCherry expression driven by the *rab-3* promoter. Similar changes were observed in another independent line. Expression was highest in the posterior intestine and generally became visible around Day 5 of adulthood and was maintained at least until Day 10. Scale bars, 200 μm.
(EPS)

S2 Fig Dietary-restricted *eat-2* mutants display tissue-specific differences in the GFP:LGG-1 autophagy reporter. Representative confocal fluorescence images of *eat-2* animals (**A, C, E**) and quantification (**B, D, F**) of GFP::LGG-1 punctae (arrows) in intestine (**A, B**), body-wall muscle (**C, D**), and neurons (**E, F**) of wild-type (WT) *C*. *elegans* and *eat-2(ad1116)* mutants fed OP50 bacteria and analyzed on Day 3 of adulthood. For the intestine, the published *lgg-1p*::*gfp*::*lgg-1* reporter was used, and punctae were counted in one 0.6 μm Z-stack per cell per animal as indicated by the dashed lines in (**A**). Error bars are very tight in (**B**) and are therefore not visible. For body-wall muscle, the same *lgg-1p*::*gfp*::*lgg-1* reporter was used, and punctae were counted in a 1000 μm^2^ area where muscle striations were most visible (one 0.6 μm Z-stack per animal). For neurons, a newly developed *rgef-1p*::*gfp*::*lgg-1* reporter was used (see Methods), and punctae were counted in all neurons located between the two pharyngeal bulbs (dashed lines in (**E**); one 0.6 μm Z-stack per animal). Data are the mean ± SEM of ~20–50 animals per condition from three independent experiments. ****P<0.0001 and n.s., not significant, Student’s *t*-test. Scale bars, 20 μm.
(EPS)

S3 Fig The intestine of 7-day-old dietary-restricted *eat-2* mutants also display a reduced number of GFP::LGG-1 punctae and an increase in autolysosomes. (**A**) Quantification in confocal fluorescence images of GFP::LGG-1 punctae in the intestine of *eat-2(ad1116)* mutants fed OP50 bacteria on Day 7 of adulthood. Quantification was done using an *lgg-1p*::*gfp*::*lgg-1* reporter and punctae were counted in one 0.6 μm Z-stack per cell per animal as indicated by the dashed lines in S2A Fig. Data are the mean ± SEM of ~20–50 animals per condition from three independent experiments. ****P<0.0001, Student’s *t-*test. Error bars are very tight and are therefore not visible. (**B**) Quantification of autolysosome-like structures in electron micrographs of intestinal cross-sections in *eat-2(ad1116)* animals raised on OP50 on Day 7 of adulthood. Data are the mean ± SEM of 14–20 micrographs. *P = 0.013, Student’s *t*-test.
(EPS)

S4 Fig The age-related breakdown of intestinal barrier function is reduced in insulin-signallng *daf-2* mutants and autophagy gene knockdown reduces the intestinal integrity of *eat-2* mutants. (**A**) Quantification of body-cavity leakage in *daf-2(e1370)* mutants raised on OP50 bacteria. Animals were examined after soaking in dye for 3 h on the indicated days of adulthood (see Methods). Data are the mean ± SEM of three biological repeats per time point, each with 8–10 animals. *P = 0.027 by Student’s *t*-test. (**B**) Quantification of body-cavity leakage in wild-type (N2) animals fed from Day 1 of adulthood with bacteria containing empty vector (control) or expressing *atg-18/Wipi*-targeting dsRNA. Data are the mean ± SEM of three biological repeats per time point, each with 8–10 animals. (**C, D**) Quantification of body-cavity leakage in 15-day-old *eat-2(ad1116); rde-1(ne219)* mutants expressing *rde-1* from the *nhx-2* promoter fed from Day 1 of adulthood with bacteria containing empty vector (control) or expressing *vha-15* (**C**) or *lgg-1+lgg-2* mixed (**D**) targeting dsRNA. Data are the mean ± SEM of three biological repeats, each with 8–10 animals. *P = 0.028 by Student’s *t-*test for *vha-15* RNAi.
(EPS)

S5 Fig Wild-type and *eat-2* animals display a similar age-related decline in motility. Body-bend rates of wild-type (WT, N2) and *eat-2(ad1116)* animals. Data are the mean ± SEM of 6 animals per time point. *P = 0.02, Student’s *t*-test. The experiment was repeated three times with similar results.
(EPS)

S6 Fig Unlike WT animals, *eat-2* mutants show no visible abnormalities in myosin expression upon autophagy gene knockdown. (**A**) Representative confocal images of 15-day-old wild-type (WT) and *eat-2(ad1116)* animals expressing *myo-3p*::*gfp*::*myo-3* and fed from Day 1 of adulthood with bacteria containing empty vector (control) or expressing *atg-18*/WIPI-targeting dsRNA. All animals subjected to *atg-18* RNAi displayed an uncoordinated phenotype (reflecting mobility decline). The experiment was repeated twice with similar results. Arrows indicate examples of sarcomere deterioration events. (**B**) Quantification of sarcomere deterioration events (using a slightly modified protocol from Zhang *et al*., 2013, see Methods). N = 6–8 for WT, and N = 10 for *eat-2(ad1116)*. *P = 0.021, Student’s *t-*test.
(EPS)

S7 Fig Muscle-specific autophagy gene knockdown decreases the lifespan of dietary-restricted *eat-2* mutants. Lifespan analysis of *eat-2(ad1116)* single mutants and *eat-2(ad1116); sid-1(qt9)* double mutants expressing *sid-1* from the *myo-3* promoter. Animals were fed bacteria expressing either empty-vector control or dsRNA encoding *atg-18*/*WIPI* from Day 1 of adulthood. Dietary-restricted *eat-2* single mutants were subject to whole-body RNAi, whereas *eat-2; sid-1* mutants carrying transgenes were subject to tissue-specific RNAi. All experiments were carried out at 20°C and were performed at least three times with similar results. Autophagy was inhibited using *lgg-1/lgg-2/ATG8* RNAi rather than *atg-18/Wipi* RNAi in at least one replicate experiment. See **S1 Table** for details and additional experiments.
(EPS)

S1 Table Lifespan analysis of *eat-2* mutants subjected to tissue-specific RNAi against autophagy genes. Lifespan analysis of *eat-2(ad1116)* mutants capable of tissue-specific RNAi. Specifically, *eat-2(ad1116); rde-1(ne219)* double mutants carrying the intestine-specific promoter (*nhx-2*)-driven tissue-specific arrays were used to re-establish RNAi in the intestine. Moreover, *eat-2(ad1116); sid-1(qt9)* mutants carrying tissue-specific arrays to re-establish RNAi in a particular tissue were analyzed (intestinal *gly-19* promoter, body-wall muscle promoter *myo-3*, and neuronal *rab-3* promoter, see **S1 Fig** and Methods for notes on *rab-3* promoter leakage). Animals were incubated at 20°C and fed from Day 1 of adulthood with bacteria containing empty vector (control) or expressing dsRNA targeted to the indicated autophagy genes (RNAi). RNAi of *lgg-1* and *lgg-2* has a synergistic effect on lifespan reduction [55] and RNAi clones for these genes were therefore used together. Of note, autophagy gene RNAi had no effect on the lifespan of control *eat-2(ad1116); rde-1(ne219)* or *eat-2(ad1116); sid-1(qt9)* double mutants not containing tissue-specific arrays. Lifespan analysis of WT and *eat-2(ad1116)* animals subjected to whole-body RNAi was performed in parallel with these experiments. Although five representative examples are shown in this table, >20 experiments were performed testing whole-body *eat-2* RNAi with similar results. ^1, 2^ indicate the experiments in which WT control animals were examined in parallel with *eat-2(ad1116)* mutants. The table shows the mean lifespans of controls and animals subjected to RNAi (avg lifespan), percent change in the mean lifespan for RNAi versus control, *P* value for the difference between RNAi and control calculated by the Mantel-Cox log-rank test, and the number of animals (number of dead animals subjected to RNAi/total number of animals analyzed). ^ indicates that this *eat-2(ad1116); sid-1(qt9)* double mutant was derived from a further outcrossed *sid-1(qt9)* strain (see **S2 Table**). * indicates the data shown in **Fig 1**.
(DOCX)

S2 Table *C*. *elegans* strains used in this study. (DOCX)

S3 Table Sequences of quantitative RT-PCR primers used in this study. (DOCX)
