# Correction: Intestinal Autophagy Improves Healthspan and Longevity in *C*. *elegans* During Dietary Restriction

**DOI:** 10.1371/journal.pgen.1006271

**Published:** 2016-08-16

**Authors:** Sara Gelino, Jessica T. Chang, Caroline Kumsta, Xingyu She, Andrew Davis, Christian Nguyen, Siler Panowski, Malene Hansen

Fig 1A is incorrectly cited instead of Fig 2A in the subsection ‘The intestine of *eat-2* mutants contains an enlarged acidic compartment and displays accelerated autophagosome turnover’ of the Results section. The sentence ‘RNAi from the L4 larval stage to Day 4 of adulthood caused a ~4-fold accumulation of GFP::LGG-1 punctae in the intestine of *eat-2(ad1116)* mutants whereas a small, but insignificant ~5% increase was observed in WT animals (Fig 1A).’ should read: ‘RNAi from the L4 larval stage to Day 4 of adulthood caused a ~4-fold accumulation of GFP::LGG-1 punctae in the intestine of *eat-2(ad1116)* mutants whereas a small, but insignificant ~5% increase was observed in WT animals (Fig 2A).’
