# Supplementary material for: Intestinal Autophagy Improves Healthspan and Longevity in C. elegans during Dietary Restriction
Source: PLoS Genet. 2016 Jul 14;12(7):e1006135. doi: 10.1371/journal.pgen.1006135 (PMC4945006; doi:10.1371/journal.pgen.1006135)
Supplement: S1 Table — Lifespan analysis of eat-2(ad1116) mutants capable of tissue-specific RNAi. Specifically, eat-2(ad1116); rde-1(ne219) double mutants carrying the intestine-specific promoter (nhx-2)-driven tissue-specific arrays were used to re-establish RNAi in the intestine. Moreover, eat-2(ad1116); sid-1(qt9) mutants carrying tissue-specific arrays to re-establish RNAi in a particular tissue were analyzed (intestinal gly-19 promoter, body-wall muscle promoter myo-3, and neuronal rab-3 promoter, see S1 Fig and Methods for notes on rab-3 promoter leakage). Animals were incubated at 20°C and fed from Day 1 of adulthood with bacteria containing empty vector (control) or expressing dsRNA targeted to the indicated autophagy genes (RNAi). RNAi of lgg-1 and lgg-2 has a synergistic effect on lifespan reduction [55] and RNAi clones for these genes were therefore used together. Of note, autophagy gene RNAi had no effect on the lifespan of control eat-2(ad1116); rde-1(ne219) or eat-2(ad1116); sid-1(qt9) double mutants not containing tissue-specific arrays. Lifespan analysis of WT and eat-2(ad1116) animals subjected to whole-body RNAi was performed in parallel with these experiments. Although five representative examples are shown in this table, >20 experiments were performed testing whole-body eat-2 RNAi with similar results. 1, 2 indicate the experiments in which WT control animals were examined in parallel with eat-2(ad1116) mutants. The table shows the mean lifespans of controls and animals subjected to RNAi (avg lifespan), percent change in the mean lifespan for RNAi versus control, P value for the difference between RNAi and control calculated by the Mantel-Cox log-rank test, and the number of animals (number of dead animals subjected to RNAi/total number of animals analyzed). ^ indicates that this eat-2(ad1116); sid-1(qt9) double mutant was derived from a further outcrossed sid-1(qt9) strain (see S2 Table). * indicates the data shown in Fig 1. (DOCX) [file pgen.1006135.s008.docx]

S1 Table: Lifespan analysis of *eat-2* mutants subjected to tissue-specific RNAi against autophagy genes.

| **Genotype of strain** | **Promoter expressing *sid-1*** | **Adult-only RNAi treatment** | **RNAi average lifespan (days)** | **Control average lifespan (days)** | **Percent change** | ***P* value** | **Number of animals** |
| --- | --- | --- | --- | --- | --- | --- | --- |
|  |  |  |  |  |  |  |  |
|  |  |  |  |  |  |  |  |
|  |  |  |  |  |  |  |  |
|  |  |  |  |  |  |  |  |
| **Assays using tissue-specific RNAi strains** | | | | | | | |
| *eat-2; rde-1* | *nhx-2p* | *atg-18** | 18.5 | 23.6 | -22 | <0.0001 | 58/89 |
|  |  | *atg-18* | 20.0 | 22.6 | -12 | 0.0009 | 70/120 |
|  |  | *atg-18* | 17.3 | 21.2 | -18 | <0.0001 | 69/85 |
|  |  | *lgg-1/lgg-2* | 19.4 | 21.5 | -10 | 0.0005 | 80/94 |
|  |  | *lgg-1/lgg-2** | 21.0 | 23.8 | -12 | 0.0002 | 71/98 |
|  |  | *lgg-1/lgg-2* | 21.1 | 26.3 | -30 | <0.0001 | 69/73 |
| *eat-2; sid-1* | *gly-19p* | *atg-18* | 20.7 | 29.6 | -30 | <0.0001 | 64/91 |
|  |  | *atg-18* | 17.8 | 26.3 | -32 | <0.0001 | 97/111 |
|  |  | *atg-18* | 19.4 | 26 | -25 | <0.0001 | 98/115 |
|  |  | *lgg-1/lgg-2* | 21.1 | 26.5 | -20 | <0.0001 | 90/120 |
|  |  | *lgg-1/lgg-2* | 22.9 | 27 | -15 | <0.0001 | 105/111 |
| *eat-2; sid-1* | *myo-3p* | *atg-18* | 22.6 | 27.2 | -17 | <0.0001 | 84/87 |
|  |  | *atg-18* | 20.8 | 25.1 | -17 | <0.0001 | 108/119 |
|  |  | *atg-18* | 18.9 | 26.8 | -29 | <0.0001 | 91/117 |
|  |  | *lgg-1/lgg-2* | 19.9 | 25.7 | -22 | <0.0001 | 91/105 |
| *eat-2; sid-1* | *rab-3p* | *atg-18* | 20.9 | 23.7 | -12 | 0.004 | 52/74 |
|  |  | *atg-18* | 21.1 | 24.4 | -13 | <0.0001 | 57/88 |
|  |  | *atg-18* | 18.1 | 26.1 | -30 | <0.0001 | 97/111 |
|  |  | *lgg-1/lgg-2* | 20.4 | 22.4 | -9 | 0.0063 | 107/121 |
| **Control assays using whole-body RNAi** | | | | | | | |
| WT | - | *atg-18^1^* | 21.8 | 19.6 | 11 | 0.002 | 92/104 |
|  |  | *atg-18^2^* | 14.0 | 16.9 | -17 | 0.0006 | 65/85 |
|  |  | *lgg-1/lgg-2^1^* | 20.5 | 21.5 | -5 | 0.63 | 39/113 |
|  |  | *lgg-1/lgg-2^2^* | 22.5 | 20.2 | 11 | 0.05 | 52/106 |
| *eat-2* | - | *atg-18_1_* | 17.1 | 22.9 | -25 | <0.0001 | 88/101 |
|  |  | *atg-18_2_* | 14.7 | 19.3 | -24 | <0.0001 | 70/84 |
|  |  | *atg-18* | 17.9 | 28.9 | -38 | <0.0001 | 95/119 |
|  |  | *atg-18* | 17.7 | 28.5 | -38 | <0.0001 | 75/105 |
|  |  | *atg-18* | 16.4 | 24.5 | -34 | <0.0001 | 77/102 |
|  |  | *lgg-1/lgg-2^1^* | 22.7 | 28.9 | -21 | <0.0001 | 95/114 |
|  |  | *lgg-1/lgg-2^2^* | 23.1 | 28.5 | -19 | 0.0001 | 51/108 |
|  |  | *lgg-1/lgg-2* | 16.5 | 22.2 | -26 | <0.0001 | 97/120 |

**S1 Table (continued):**

| **Genotype of strain** | **Promoter expressing *sid-1*** | **Adult-only RNAi treatment** | **RNAi average lifespan (days)** | **Control average lifespan (days)** | **Percent change** | ***P* value** | **Number of animals** |
| --- | --- | --- | --- | --- | --- | --- | --- |
|  |  |  |  |  |  |  |  |
|  |  |  |  |  |  |  |  |
|  |  |  |  |  |  |  |  |
|  |  |  |  |  |  |  |  |
| **Assays using control tissue-specific RNAi strains** | | | | | | | |
| *eat-2; sid-1* | None | *atg-18* | 28.0 | 30.4 | -9 | 0.25 | 45/69 |
|  |  | *atg-18* | 31.5 | 32.3 | -2 | 0.001 | 37/86 |
| *eat-2; sid-1^* | None | *atg-18* | 27.2 | 29.9 | -9 | 0.20 | 49/82 |
|  |  | *atg-18* | 30.1 | 29.8 | 1 | 0.92 | 49/73 |
| *eat-2; rde-1* | None | *atg-18* | 25.0 | 22.8 | 10 | 0.3 | 65/104 |
|  |  | *atg-18* | 25.2 | 28.5 | -12 | 0.007 | 38/68 |
|  |  | *atg-18* | 24.1 | 23.7 | 2 | 0.8 | 49/73 |

Lifespan analysis of *eat-2(ad1116)* mutants capable of tissue-specific RNAi. Specifically, *eat-2(ad1116); rde-1(ne219)* double mutants carrying the intestine-specific promoter (*nhx-2*)-driven tissue-specific arrays were used to re-establish RNAi in the intestine. Moreover, *eat-2(ad1116); sid-1(qt9)* mutants carrying tissue-specific arrays to re-establish RNAi in a particular tissue were analyzed (intestinal *gly-19* promoter, body-wall muscle promoter *myo-3*, and neuronal *rab-3* promoter, see **S1 Fig** and Methods for notes on *rab-3* promoter leakage).

Animals were incubated at 20°C and fed from Day 1 of adulthood with bacteria containing empty vector (control) or expressing dsRNA targeted to the indicated autophagy genes (RNAi). RNAi of *lgg-1* and *lgg-2* has a synergistic effect on lifespan reduction [23] and RNAi clones for these genes were therefore used together. Of note, autophagy gene RNAi had no effect on the lifespan of control *eat-2(ad1116); rde-1(ne219)* or *eat-2(ad1116); sid-1(qt9)* double mutants not containing tissue-specific arrays. Lifespan analysis of WT and *eat-2(ad1116)* animals subjected to whole-body RNAi was performed in parallel with these experiments. Although five representative examples are shown in this table, >20 experiments were performed testing whole-body *eat-2* RNAi with similar results. ^1, 2^ indicate the experiments in which WT control animals were examined in parallel with *eat-2(ad1116)* mutants.

The table shows the mean lifespans of controls and animals subjected to RNAi (avg lifespan), percent change in the mean lifespan for RNAi versus control, *P* value for the difference between RNAi and control calculated by the Mantel-Cox log-rank test, and the number of animals (number of dead animals subjected to RNAi/total number of animals analyzed). ^ indicates that this *eat-2(ad1116); sid-1(qt9)* double mutant was derived from a further outcrossed *sid-1(qt9)* strain (see **S2 Table**). * indicates the data shown in **Fig 1**.
