# Supplementary material for: Intestinal Autophagy Improves Healthspan and Longevity in C. elegans during Dietary Restriction
Source: PLoS Genet. 2016 Jul 14;12(7):e1006135. doi: 10.1371/journal.pgen.1006135 (PMC4945006; doi:10.1371/journal.pgen.1006135)
Supplement: S2 Table — (DOCX) [file pgen.1006135.s009.docx]

**S2 Table: *C. elegans* strains used in this study.**

| **Published strains used in this study** | | | |
| --- | --- | --- | --- |
| **Name** | **Genotype** | | **Strain obtained from** |
| N2-Hansen | Wild-type (WT) | | Hansen lab, originally from Kenyon lab |
| AGD620 | *eat-2(ad1116) III; sid-1(qt9) V;  uthEx228[myo-3p::tdtomato + myo-3p::sid-1]* | | Dillin lab |
| AGD855 | *sid-1(qt9) V; uthIs237[myo-3p::tdtomato + myo-3p::sid-1]* | | Dillin lab |
| CF1908 | *eat-2(ad1116) II* | | Kenyon lab |
| DA2123 | *adIs2122[lgg-1p::gfp::lgg-1 + rol-6]* | | Avery lab |
| HC196 | *sid-1(qt9) V* | | Hunter lab |
| RW1596 | *myo-3(st386)V; stEx30[myo-3p::gfp::myo-3+ rol-6]* | | Waterston lab |
| VP303 | *rde-1(ne219) V; kbIs7[nhx-2p::rde-1 + rol-6]* | | Strange lab |
| WM27 | *rde-1(ne219) V* | | Mello lab |
| **New strains used in this study** | | | |
| **Name** | **Genotype** | **Comments** | |
| MAH13 | *rde-1(ne219) V* | WM27 outcrossed 4x | |
| MAH34 | *eat-2(ad1116) II; adIs2122[lgg-1p::gfp::lgg-1 + rol-6]* | CF1908 x MAH13 | |
| MAH51 | *eat-2(ad1116) II; rde-1(ne219) V* | MAH13 x CF1908 | |
| MAH64 | *eat-2(ad1116) II; rde-1(ne219) V; kbIs7[nhx-2p::rde-1 + rol-6]* | MAH51 x VP303 | |
| MAH95 | *eat-2(ad1116) II* | CF1908 outcrossed 4X | |
| MAH185 | *sqEx21[rgef-1p::gfp::lgg-1 + unc-122p::rfp]* | Injection into N2 | |
| MAH230 | *sqIs24[rgef-1p::gfp::lgg-1 +unc-122p::rfp]* | Gamma-irradiation of MAH185 | |
| MAH242 | *sqIs24[rgef-1p::gfp::lgg-1 +unc122p::rfp]* | MAH230 outcrossed 4x | |
| MAH259 | *eat-2(ad1116) II*; *sqIs24[rgef-1p::gfp::lgg-1 + unc-122p::rfp]* | CF1908 x MAH242 | |
| MAH317 | *eat-2(ad1116) II*; *myo-3(st386) V ?;  stEx30[myo-3p::gfp::myo-3+ rol-6]* | HC196 outcrossed 4x | |
| MAH338 | *sqEx36[ab-3p::mCherry +rol-6]* | Injection of Addgene construct | |
| MAH339 | *sqEx37[rab-3p::mCherry +rol-6]* | Injection of Addgene construct | |
| MAH346 | *sid-1(qt9) V* | HC196 outcrossed 4x | |
| MAH382 | *eat-2(ad1116) II; sid-1(qt9) V* | MAH95 x AGD620 | |

**S2 Table (continued):**

| **New strains used in this study** | | |
| --- | --- | --- |
| **Name** | **Genotype** | **Comments** |
| MAH406 | *eat-2(ad1116) II; sid-1(qt9) V* | MAH95 x MAH346 |
| MAH458 | *eat-2 eat-2(ad1116) II* | MAH95 outcrossed 2x |
| MAH494 | *eat-2; sid-1(qt9); alxIs6[vha-6p::sid-1::sl2::gfp]* | MAH406 x MAH462 |
| AGD567 | *eat-2(ad1116) II; sid-1(qt9) V* | HC196 x DA1116 |
| AGD568 | *eat-2(ad1116) III, sid-1(qt9) V;  uthEx236[gly-19p::tdtomato + gly-19p::sid-1]* | Extrachromosomal array, injected into AGD567 |
| AGD631 | *eat-2(ad1116) III; sid-1(qt9) V; uthIs201[rab-3p::sid-1 + rab-3p::tdtomato]* | Injection into AGD567 and integrated, outcrossed 1x |
| AGD635 | *sid-1(qt9) V; uthEx236[gly-19p::tdtomato + gly-19p::sid-1]* | Derived from AGD568 by crossing to HC196 |
| AGD745 | *sid-1(qt9) V; uthIs236[gly-19p::tdtomato + gly-19p::sid-1]* | AGD635 integrated and outcrossed 7x |
| AGD803 | *eat-2(ad1116) II; sid-1(qt9) V;  uthIs236[gly-19p::sid-1 + gly-19p::tdtomato]* | AGD745 x AGD567 |
| AGD973 | *eat-2(ad1116) II; sid-1(qt9) V;  uthIs237[myo-3p::sid-1 + myo-3p::tdtomato]* | AGD855 x AGD567 |
