# Supplementary material for: Intestinal Autophagy Improves Healthspan and Longevity in C. elegans during Dietary Restriction
Source: PLoS Genet. 2016 Jul 14;12(7):e1006135. doi: 10.1371/journal.pgen.1006135 (PMC4945006; doi:10.1371/journal.pgen.1006135)
Supplement: S3 Table — (DOCX) [file pgen.1006135.s010.docx]

**S3 Table. Sequences of quantitative RT-PCR primers used in this study.**

| **Gene** | **Primer sequence 5’ 🡪 3’** | |
| --- | --- | --- |
| **Experimental genes** | | |
| *atg-18* | Fwd | AAA TGG ACA TCG GCT CTT TG |
|  | Rev | TGA TAG CAT CGA ACC ATC CA |
| *lgg-1* | Fwd | acc cag acc gta ttc cag tg |
|  | Rev | acg aag ttg gat gcg ttt tc |
| *sqst-1* | Fwd | TGG CTG CTG CAT CAT CCG CT |
|  | Rev | TCA ATC GTG CCG AGA CCG GG |
| *vps-11* | Fwd | TCC GCT TGT CGT CCT GGA GC |
|  | Rev | TCA CAC GCC GAG CAC TTG GT |
| *vha-15* | Fwd | CGA GGT TCG TTC CGG ACG TCT T |
|  | Rev | CCT CGG CAG TCA GGA GAC GC |
| *vha-16* | Fwd | AGG CGC TGA CTC GCG GAC TT |
|  | Rev | TGG TCT CTG GTG AAG AGT TCC GGT G |
| **Housekeeping genes** | | |
| *ama-1* | Fwd | TGG AAC TCT GGA GTC ACA CC |
|  | Rev | CAT CCT CCT TCA TTG AAC GG |
| *nhr-23* | Fwd | CAG AAA CAC TGA AGA ACG CG |
|  | Rev | CGA TCT GCA GTG AAT AGC TC |
